# Sickle cell, habitual dys-positions and fragile dispositions: young people with sickle cell at school

**DOI:** 10.1111/j.1467-9566.2010.01301.x

**Published:** 2011-03

**Authors:** Simon M Dyson, Karl Atkin, Lorraine A Culley, Sue E Dyson, Hala Evans

**Affiliations:** 1 Unit for the Social Study of Thalassaemia and Sickle Cell, De Montfort UniversityLeicester; 2 Department of Health Sciences, University of York; 3 Faculty of Health and Life Sciences, De Montfort UniversityLeicester; 4 School of Nursing and Midwifery, De Montfort UniversityLeicester

**Keywords:** sickle cell, Bourdieu, chronic illness, school health, habitus

## Abstract

The experiences of young people living with a sickle cell disorder in schools in England are reported through a thematic analysis of forty interviews, using Bourdieu’s notions of field, capital and habitus. Young people with sickle cell are found to be habitually dys-positioned between the demands of the clinic for health maintenance through self-care and the field of the school, with its emphases on routines, consistent attendance and contextual demands for active and passive pupil behaviour. The tactics or dispositions that young people living with sickle cell can then employ, during strategy and struggle at school, are therefore fragile: they work only contingently, transiently or have the unintended consequences of displacing other valued social relations. The dispositions of the young people with sickle cell are framed by other social struggles: innovations in school procedures merely address aspects of sickle cell in isolation and are not consolidated into comprehensive policies; mothers inform, liaise, negotiate and advocate in support of a child with sickle cell but with limited success. Reactions of teachers and peers to sickle cell have the enduring potential to drain the somatic, cultural and social capital of young people living with sickle cell.

## Introduction

This article examines the experiences of young people living with a sickle cell disorder (SCD) in schools in England. SCDs are severe, inherited chronic illnesses, primarily associated with minority ethnic communities. The complex interaction of active engagement in shaping their world, while that world is structured historically, economically and in racist terms, is best understood, we argue, through an application of the work of Pierre Bourdieu. We use Bourdieu to explore the discursive space in which young people with sickle cell have to make sense of their lives. We first outline our justifications for using Bourdieu’s concepts of habitus, capital and field with regard to young people living with SCD. We then provide an account of the methods before providing an analysis that explores how young people with SCD negotiate in complex and nuanced ways through the fields of the clinic and the school.

## Bourdieu: habitus, capital and field

The work of Bourdieu has long informed education research, especially with respect to the cultural elements of family life that facilitate concordance with school expectations (Lareau and Horvat 1999), the impact of parental ‘choice’ of school in the reproduction of disadvantages (Ball *et al.* 1996) and the role of the teacher in reproducing practices excluding Black pupils (Lipman 1998: 158–9). Only more recently has it underpinned sociologies of health (Béhague *et al.* 2008, Brown *et al.* 2008). It has not been utilised in connection with chronic illness in schools, though there seem to be a number of theoretical reasons supporting such an approach.

In using habitus as a conceptual research tool, Bourdieu deals predominantly with habitus associated with class. However, it could and should also be applied with respect to gender and ‘race’ (Reay 2004a). In this context, habitus seems to be especially apposite for analysing the experiences of living with SCD, since sickle cell has attained the status of being an ‘emblematically black’ disease (Hall 2003: 6). A child with SCD acquires particular symbolic meanings in low-income families experiencing racism (Hill 1994), and this meaning is gendered (Hill and Zimmerman 1995). In addition these meanings run counter to the stereotype that Black people embody high degrees of physical capital (Fleming 2001). Habitus may be understood as a putative resolution to the sense of an individual overdetermined by their culture, as well as the equally problematic proposition that agency could be independent of structure. Habitus is the collection of habitual orientations that individuals come to use in navigating their lives. Health experience cannot be assumed from the condition’s severity; young people with sickle cell are active in negotiating their illness world and engage in various strategies to legitimate their experience (Atkin and Ahmad 2001). This suggests that cultural habitus may be a profitable concept within which to examine experiences of young people living with SCD at school.

Bourdieu identifies the school as a key institution in the generation of cultural capital (Bourdieu and Passeron 1977), the source not only of formal educational qualifications but also of generating the right habits and tastes, valued ways of speaking, and ways of being in the world (Bourdieu 1984). Economic capital is important in indirectly buying educational opportunity through the purchase of homes within the catchment areas of desirable schools (Demaine 2006). It is also contended that Black students in English schools fall further behind the longer they are in the education system (Gillborn 2008), and economic capital is used by some Black families in an attempt to stem or reverse this negative capital flow by use of Black supplementary schools. Since Bourdieu’s initial reformulation of the concept of capital (Bourdieu 1997) it has been extended further, for example to include somatic capital. Whiteness is an unacknowledged symbolic resource (Atkin 2004). Of the types of capital ‘allowed’ to Black pupils (artistic and sporting) the latter is relatively unavailable to those living with SCD.

In addition our bodies have become our life projects (Giddens 1991) and this assumes a particular significance for children who have a long-standing health condition. What potentially connects capital to habitus is that by maintaining their habitual preventive behaviour of keeping warm, being well hydrated and avoiding strenuous exercise and stress, young people with SCD can build up health capital that may prevent or reduce key disease events such as painful sickle cell crises. This provides them with strategies, as they struggle to exercise some control over their condition, though these are only strategies, being handed down from and sanctioned by medical power, as distinct from tactics, the more contingent local navigations of lived experience (de Certeau 1984). Such strategies also imply responsibility: an increasing feature of modern medical discourse (see Gately *et al.* 2007).

What connects Bourdieu’s notion of capital to the final part of his conceptual map, field, is that the school is the most significant field in which cultural capital can be accumulated. In Bourdieu’s terms a field is a structured space of (a struggle for) positions. A school combines a bounded physical space, structured positions (teacher and pupil) and internal (setting) and external (league tables) hierarchies. It is an arena of strategies and struggles, informed by notions of racism from the very outset (Youdell 2003). The school-as-field has a degree of autonomy from the outside society, having its own social relations, hidden curriculum and school ethos (although such structured social relations cannot entirely divorce themselves from broader societal norms and values). Schools also represent a particular historical constellation or configuration. As Tomlinson (2008) shows, eras of assimilation and accommodation have structured the experience of Black pupils in schools. This is not to say that the school is the only discernible field within which young people with SCD struggle. They are part of a community field and perhaps additionally a separate Black community field to the extent that residential, economic and cultural segregation prevail.

They are also part of a field we might call, after Foucault (1973), the clinic (Hudson 2008), in which regular outpatient checks and tests, in-patient stays for treatment of acute episodes and for prevention of strokes take up a considerable proportion of their time. But the medical gaze now extends into the community (Armstrong 1983) and technologies of the self (Foucault 1988) produce the expert patient who self-monitors health for the greater good not only of their own somatic capital but for the benefit of the broader health economy (Pescosolido 2006). What happens, however, when the field of the school has to connect with the field of the clinic? And in particular how do young people negotiate this?

## Conducting the research

The fieldwork was undertaken using a multi-method study of the experiences of young people with SCD in schools in England (Dyson *et al.* 2010a). Approval was obtained from a multi-centre ethics committee. Signed informed consent was obtained from the respondents and their main carer. A total of 40 taped interviews were conducted by one of three different research team members with young people, mainly of secondary school age and young adults (see Table 1).

**Table 1:** Characteristics of the interviewees (n), total = 40

Gender	
Female	21
Male	19
Age	
5–10	2
11–18	30
19–25	8
Ethnicity	
Black African	24
Black Caribbean	15
British Asian (Indian)	1
Venue of interview	
Hospital out-patients	21
Community centre	11
Home	3
Counselling centre	3
University	2
Present at interview	
Young person only	33
Young person plus mother	5
Young person plus father	1
Young person plus counsellor	1

The interviews were transcribed in full by the main interviewer (HE) or by one of two transcribers. The first 15 interviewees were selected opportunistically in conjunction with the administration of a questionnaire study in which 569 respondents were indentified through 12 local support groups, three sickle cell counselling centres or three outpatient clinics. The questionnaire invited volunteers, and 200 potential interviewees were identified. All authors reviewed the transcripts of the first 15 interviews and, in the spirit of the open stage of theoretical sampling (Glaser and Strauss 1967), suggested further sampling that would provide greater diversity in experiences. The first round of interviews produced predominantly negative accounts of experiences. The subsequent sampling strategy was to identify five people with SCD who were succeeding (defined as being likely to or actually attending university) and five who had left school (or whose school trajectory suggested they were likely to do so) at the minimum school leaving age. This strategy produced a further 10 interviewees. We also used the information from the questionnaires to inform the sampling. One item invited respondents to assess the extent to which they thought they had been helped to catch up on lessons missed. The five points of the scale (0, 25, 50, 75 and 100%) were used to identify five male and five female respondents, one for each of these varied experiences. We return to consider this variation in experience later in considering who gains educational capital and why.

The transcripts were reviewed by all five authors and each suggested a list of themes. A meeting of the authors found no fundamental disagreement in themes, although they were expressed differently (see Armstrong *et al.* 1997). The one exception was how to regard the single explicit reference to racism that occurred in the interviews. This was addressed by incorporating a specific question on racism into the interview schedule for a subsequent stage of research that included interviews with the original respondents’ parents, in order to explore the comparative absence of explicit references to racism.

Our analysis attempts to move beyond a descriptive account of specific interviews towards a level of analysis in which themes were explicated in existent and emergent theory. In writing up qualitative research, there are no particular rules to suggest how many themes can best represent the diversity of a dataset (Reissman 2008). In our case, we specifically assumed an intersubjective notion of identity, which was used to construct a series of themes, reflecting the respondents’ validation of who they were, selected purposively to engage with our general theoretical argument. We did not reduce our respondents’ remarks to expressions of Bourdieusian theory but used social theory to highlight tensions in how those with SCD experience everyday life.

A total of 20 main themes were constructed from the transcripts. Their frequency was mapped (see Table 2) and the themes were conceptualised under four main headings: the field of the clinic (health behaviour deemed necessary to maintain good health and challenges to implementing them); the field of the school (school absences, school re-entry, catching up lessons and seeking support); the habitus of the young person with SCD (managing interpersonal relations at school) and contextual factors including the role of the mother; and the potential for reactions to SCD to be a drain on the respondents’ resources and the relatively rare innovations in school practices that responded to their needs. An attempt to represent a relationship between these factors is given in Table 2 and discussed further below.

**Table 2:** Representation of key themes of interviews with young people with sickle cell disorder

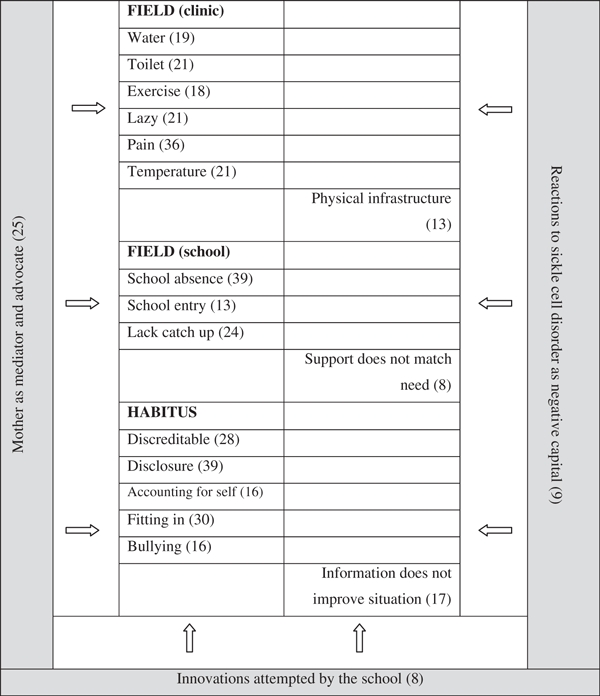

## The field of the clinic: technologies of living with SCD

The reflexivity required by Bourdieu (Bourdieu and Wacquant 1992) compels us to acknowledge that the themes listed under the field of the clinic are essentially themes introduced by direct questioning by the research team based on existing literature (Anionwu and Atkin 2001) and the voluntary work experience in sickle cell of two research team members (who were thereby cognisant of issues perennially raised in self-help settings). The themes also reflect the power of the clinic (Foucault 1973) in that the most successful recruitment route producing subjects for the study was through out-patient clinics. Other reflexivities, on the community of sociology and the dangers of scholastic viewpoint (Kenway and McLeod 2004) lead us to acknowledge the irony that the gravitas of Bourdieu (see Reay 2004a) has become a condition in establishing the voice of young people with SCD within a forum of elite knowledge, in order to reduce the possibility that policymakers, like teachers, ‘didn’t believe it’, ‘didn’t listen’ and ‘just ignored it’.

In referring to the field of the clinic, we mean not just the physical space but the self-regulatory governance that is inculcated in young people with SCD: keeping healthy by drinking water, avoiding strenuous exercise, resting when tired, keeping warm and dry, staying alert to possible triggers to painful sickle cell episodes and initiating early self-treatment (Oni *et al.* 2006), which are all indicators that SCD is not a given but that the physical capital embodied in someone with SCD can in principle be ameliorated,. This does sometimes create tensions within the field of the school which young people have to negotiate, using various tactics, with varying degrees of success. The field of the school, in effect, embodies notions of acceptable behaviour that are different from those expected by more medically orientated discursive practices, while also reproducing expected norms of body in terms of gender, disability and racism (Shilling 2004).

People with SCD are advised to remain well hydrated in order to reduce the likelihood of illness episodes, but this was not always permitted at school. One young woman recounted:

G8: I had to get water when I was in class. I told my teacher but she said I should have got water from the nurse at lunchtime and, and also there’s a [warning score] it says you can’t go to get a drink during class time. (Female, 13, Black African)

Furthermore, even without this additional liquid, people with SCD cannot concentrate urine as readily and need to go to the toilet frequently. The mother of a 6-year-old boy reported:

B2: There is also a second time when he went to school, my daughter went to pick him up, he was all wet in his pants. I was upset and said, ‘No, the teacher should know that this child has sickle cell, sometimes he needs help, he is a sick child, and of course he needs help’. He wet his pants and stayed wet until my daughter picked him up. (Mother of Male, 6, Black African)

The notion that drinks and toilet breaks are perceived to disrupt school routines appears to be confirmed by B4 (Male, 16, Black African), who reported that the teachers ‘said it was like a way of preparing you for secondary school’, a restriction presented to him as helping the young pupils prepare for the greater discipline that will be required of them when they progress to secondary education. Although restrictions on clinical ideals of self-care were not experienced by all respondents (see Table 2), nor indeed all of the time, teachers who demonstrated practical care consistently had to overcome the contingencies of school routines in order to do so.

People with SCD are advised to avoid strenuous exercise, but in school there appears to be an implicit expectation that pupils will engage in exercise to their physical limits:

B11: My teacher, I was in PE class and like, he was keep on, like, pushing me, pushing me, and I didn’t like much, football, and I’d tell him, like, I didn’t like doing and, like, when I was sitting out he’d come up to me: ‘Stand up’ and, like, ‘Play the game’. (Male, 15, Black Caribbean)

The young man suggests that he was sitting out and taking a rest, as the clinical mentality expects of him, in order not to provoke a sickle cell crisis. The teacher’s response was possibly overlaid with gendered and racialised expectations, namely that boys should like football or that Black pupils should excel at sport. The final phrase has connotations of habitus inculcated on the playing fields of Eton. The pupil, in resisting participation in sports (which might trigger illness), is literally sitting out the transmission of sport-related cultural capital, though in any case only ‘the symbolic value of upper-class bodies can be converted into social and cultural capital’ (Shilling 2004: 478). This renders the stance of the teacher, positioning the pupil as somehow not being fair to the teacher, who has a responsibility to ensure participation in sports values by all pupils, a double symbolic insult.

A key clinical symptom of SCD is chronic anaemia, leaving the person lethargic and unable to concentrate. A young woman recalls being tired in association with her SCD:

G1: the teacher is ‘[Name], why is your arm on the table? Why are you so lazy?’ You know, it’s like I’m not lazy, I’m just tired. And it just got to a point where I was so tired I couldn’t be bothered to argue so I just walked out the class. And then the teacher came: ‘Why did you leave my class?’ (Female, 16, Black African)

Her bodily comportment reflects the cultural arbitrariness (see Moore 2004) of the school field (sitting up straight = achieving student; slouching = under-achieving student) and disrupts general school expectations, though concordant with racist stereotypes of Black pupils as lazy (Tikly *et al.* 2006: 59). In particular, the bodily disposition of Black students and school expectations can collide unless culturally read by teachers (Youdell 2003). Moreover, her tactic, removing herself from the scene of conflict, is itself discordant with school routines. A further tactic she reports is to make use of any bursts of energy to attempt to answer questions and engage the teacher, but this is then construed as disruptive attention-seeking.

Young people with SCD are advised to maintain an optimum temperature and, in particular, to keep warm. This involves them in disputes in the face of being turned outside during break-time to face snow and rain, in resisting physical exercise classes in inclement weather and avoiding swimming in unheated pools. It also implicates them in a struggle with school uniform policies or other expectations of appropriate school attire, contextual to time and place.

G7: We weren’t allowed our coats on … I remember that I started to cry ’cos I really wanted my coat, I am not going to freeze. Everyone else was cold, I wasn’t making a big an issue out of it, ’cos I remember everyone else around me was cold, even the people who came with us were, like, ‘Yeah, it is a bit cold, these are young kids’. And they was, like, ‘No, one more time around’. And I stopped, I refused, I just could not be bothered, I just could not. I went mad, my fingers were just numb, everything was numb and I started to cry and I just felt a sharp pain, and then it all went downhill from then ’cos I had, my face was covered in gold paint and I started to cry and everything just kept running and I got sent home after that.[*Interviewer: I was going to say, how did the teachers react to?*]

G7: It was, they were just like, ‘Oh it’s not that cold’. I remember one teacher, she didn’t like me very much, she was, like, ‘It’s not that cold, oh suck it up’. I was like, ‘I am not sucking it up, it’s cold, I want my coat’,… I got really numb and I got really still and then I just started to cry. I was in one spot crying real hard and I just felt this pain and a half. (Female 18, Black African)

In this instance G7 recalls her part in the production of a school play in a cold, draughty hall while wearing a flimsy costume. Her distress was minimised by the teachers, and she was called a drama queen, arguably a gendered assumption that girls with chronic illness should be valiant, whereas boys can be vulnerable (Hill and Zimmerman 1995). However, the episode eventually triggered a painful sickle cell crisis.

What is apparent is just how inimical the routines of school are to accommodating students’ wariness of potential illness and diligence in self-care. Requests for water or toilet breaks are potentially construed as disruptive of school routines. They also undermine teachers’ attempts to develop a sense of self-discipline in the face of entering secondary school, where even greater adherence to routines that make school life possible is required. Furthermore, what connects (or perhaps more accurately what dys-connects) the field of the clinic from the field of the school are the barriers to self-care that are enshrined in the very physical provenance of the school: the dirtiness of school toilets, the unhygienic state of water fountains, the shabby school fabric with resulting extremes of temperature, the unyielding school furniture and the unpalatable school meals were all cited as contributing to the school physical environment as an unsafe place for a person with SCD.

## The field of the school: habitual dys-positions

The school is not merely a physical entity but also an arena of moral scrutiny. This takes the form of two types of monitoring, both of which, if left unmodified, undermine the accumulation of educational capital of the person with SCD. One is the league table of examination results. B16 (Male, 15, Black African) recounts that being granted a toilet break ‘depends on the teacher and the seriousness of the topic. If it is something that has to go into the GCSE results then you cannot go’. The other is monitoring of attendance. B7 (Male, 15, Black African) speculates why his school does not allow toilet breaks and drinks breaks: ‘I think I know why they don’t do that, because they’re trying to prevent truancy in the school’.

The morning roll-call is a defining part of the field of the school, and identity is inscribed through the very word for recording attendance: being *marked* absent or present. All the accounts featured commentaries on school absences associated with SCD-related illness and a minority note how lucky they were not to be more greatly affected by their SCD.

[*Interviewer: Now these pain crises you told me about in school and this feeling of tiredness, did this affect your education at this time?*]

G6: Well, yeah, because it is really hard to take it in, even now I feel struggling with it. Like, you try to listen, like, you don’t feel alert and your body is aching and it is just that you don’t really learn, you cannot take it in, it is really hard. I mean, it is hard in school, where school is. Because of my attendance, they also put me in certain subjects and what is it, I have like one, two, one three, like groups of, say, when you do exams you can just get from C to E; you cannot get higher than C. You can only receive that grade because that is what they think you are capable of and if I have the support from whatever, I reckon I could be, like, in the X band which is higher than the Y band, and you got Y and Y4 and stuff like that. And I was always put in Y2, 3 are for special needs’ kids and stuff like that. And I did not see myself, I am not saying level, so in that, there are kids there because they have special needs or whatever. And the teacher would not bother teaching them because they say ‘the longer it takes to do what you lots do because I am still getting paid. It’s up to you if you want to learn’. So that knocked me because I was put in that group work where you received Cs and Ds and Es in my exams and in my actual coursework I got As and Bs and I thought if I have the opportunity I think I would be different. Because of my attendance, because I was not in that school as much because I was sick, they just put me in that group.… They told me of course that what I would like to do, I put into (design), that was not an interest of mine. I like child care and probably food and hygiene or something like that, but they chose that for me, just because I was ill and did not have time to sit with me and give me the nurturing I needed … yeah, that was one of the biggest blows in school. (Female, 24, Black Caribbean)

In this extract, the young woman comments on how pain and anaemia associated with her condition undermined learning even when she was present. Although she achieves strongly (as evidenced by coursework results) she indicates that her school absences impacted adversely on her success. However, she suggests that this was not a direct cause, but was mediated through other, arguably racialised, relationships: that she was placed in lower bands (where teachers’ lower expectations might depress her actual achievements), associated with children with special needs (draining educational capital, though without concomitant access to modest special needs funding) and, as Gillborn (2008) has charged is the case more widely with Black pupils, reduces her educational cultural capital through entry into second-tier exams where higher attainment is precluded.

The process of returning to school after an absence also featured in the interview accounts. Returning to school entails not only missed academic lessons but missed social interaction with school peers, diminishing social capital:

G6: There is pain and that is hard to take things in because being in pain and in being in school, I find it hard. Maybe I was not with the crowd in school, that sort of thing, because [I] was not in class last week, they don’t include you. That was a quite lonely [experience] actually in school. (Female, 24, Black Caribbean)

G6 proposes that her school absence may be a reason why she is not included by her school peers and feels isolated as a result. There is also a hint that the pain and bouts of illness associated with her SCD makes ongoing inclusion challenging even when she is present at school.

The corollary of school absence is the extent to which young people with SCD felt they were helped to catch up on lessons missed. One student mentioned a learning advice centre and another one-to-one tuition from a teacher but support rarely went beyond the provision of worksheets and then sometimes only when students pressed for them. Those who claimed ‘they did not do anything for me’ included B14 (Male, 17, Black African) who in the exams ‘was the only one who did not remember because I was not there when they learnt it’. He suggests not only did school absence have a direct impact on his learning, but that he did not even comprehend what he had missed until it was revealed by his lack of ability to perform in examinations in comparison to his peers. Moreover, school presence inculcates a disposition that can convert even minimal educational capital into economic capital, for, in the absence of distinguishing features among the unqualified, regular school attendance signifies future workplace discipline.

The episodic nature of school absences associated with SCD does represent a challenge, but the lack of structured, teacher-led support to make up for lost school lessons was widely and keenly felt. We have seen how the routines of the school are in tension with the proscriptions of the clinic for self-care. Merely being present at school, integrated into peer groups and remaining connected to the required levels of work is difficult. Whatever tactics, or in Bourdieu’s term, dispositions, are available to young people living with SCD at school, they are severely circumscribed by the respective fields of the clinic and the school. This leads us to propose that young people with SCD are habitually dys-positioned, routinely positioned at an intersection of school and clinic fields that do not tessellate.

A theme linking the field of the school to the dispositions of young people with SCD is represented by the poor fit between systems of school support routinely available compared to the needs of the young person with SCD. One example of this discordance was the support of a home tutor, predicated on their absences being rare and lengthy rather than frequent but short, meaning that, by the time bureaucratic processes have been invoked and a tutor provided, the young person is back at school. A further example concerns the reactions of teachers to a painful sickle cell episode, namely summoning parents or ambulances, actions sometimes entirely appropriate. But if, in addition, the school were prepared to facilitate access to moderate painkillers and to a safe environment for time out this would often enable the students to return to classes later in the day, thereby diminishing the impact of missed schooling and of their struggles to be socially included upon re-entry to school. A third example concerns the way that teachers may misread the fact that a person with SCD continues to play as evidence that their pain is not real or has passed. Nurses routinely mistake watching TV, walking around or laughing with friends as evidence that pain is no longer felt when these activities are in fact part of a diverse range of pain-alleviation strategies (Anionwu and Atkin 2001). In short, what presages the challenge of developing tactics for living with SCD at school is that the nature of the support structures on offer is incommensurate with the nuanced requirements of the young person with SCD.

## Habitus and fragile dispositions

As well as taking account of the fields in which people conduct their social relations, we must also remember the importance of active negotiation as people make sense of who they are. The dynamic of social life, according to Bourdieu, is explained by an individual’s attempt to present a view of the world that could be regarded as legitimate by others. This requires social negotiation and is at the heart of dispositions: who you are is as much about who other people think you are as about how you define yourself (Jenkins 2008).

The corporeal nature of painful sickle cell crises certainly generated reflection in most respondents but, as opposed to the embodied social crisis of the misalignment of habitus and field (Reay 2004a), this was not experienced as a springboard to transformation. Instead, in Goffman’s (1968) terms, young people with SCD remain discreditable and vulnerable to stigmatising reactions if their SCD is revealed to others. A sudden, unpredictable onset of severe pain may reveal the young person’s SCD to peers and teachers who may not have previously known: ‘there was someone next to the ambulance listening to the conversation’ (G15, Female, 15, Black African). A recurrent feature of the interviews was the varied attitude to disclosing their SCD to others:

G17: Some understood and wanted to know what sickle cell is. Others just did not care about it and were not willing to know. Some just want to know the details, while others just turn their backs. Some said, ‘Oho, now we understand why you have these yellow eyes and you are skinny’. It was not nice to hear these comments. (Female, 16, Black Caribbean)

G17 suggests that it is the jaundice making the whites of her eyes yellow and the lower body weight associated with SCD that have made it difficult to avoid the disabling assumptions of her school peers. She also hints at our next theme: the dilemma over whether or not to disclose their SCD status to teachers or to other pupils, or both, for they may engage further or recoil from the disclosure. Indeed, one young man suggests that others may use information about their sickle cell status to deepen their discrimination against people with SCD:

B15: Because some people they can be discriminated about. So I don’t think they should teach everybody about sickle cell. This is because some people, they can discriminate against you when they learnt about it as well. (Male, 14, Black African)

The dilemma of disclosure to teachers is complex in a numbers of ways, as suggested by the following extract:

B17: Well (.) because they hear it from other people, so it is better that I tell them so I make myself not look so bad, so there are certainly some advantages and some disadvantages as well[*Interviewer: Can you give me an example, please?*]

B17: It’s, like, they make sure I am all right, I am well, if I need a drink of water. It is like I would say, ‘No, no, I am all right’. I felt that they wanted to do something and take it out of proportion. I really don’t want that (.) You know, it is hard to say no. (Male, 17, Black African)

B17 appears to say that if he initiates disclosure, then at least he has a measure of control over the presentation of self, whereas it is possible that information emanating from others may be inaccurate or be given a negative slant. Moreover, while peers may become attuned to the field of the clinic, the need to remain hydrated for example, young people with SCD do not wish to be defined only by their place in the clinical field. But neither do they wish to openly take umbrage with this positioning for fear it may damage their hard-won social capital of peer acceptance.

Irrespective of whether or not their status is known, the young people with SCD feel pressure to account for themselves (for minor somatic signs or school absences) or to explain the details of their SCD condition.

G8: Because some other students know that I’ve got sickle cell through my primary school and they’re in my secondary school in my class still and they ask me what it is sometimes. And then one day they just come up to me and say ‘Oh, what’s sickle cell? What happens?’ And stuff, and I explain, yeah. But if you tell other children that, other students that don’t know, they pass it on to other people. And then it becomes, and then they call it probably something else and they give it another description. (Female, 13, Black African)

In this account, the contrast is between those who have some history of knowing the young person and their SCD status, in which case you endure ‘a bombardment of questions that you don’t want to answer’ (B7, Male, 15, Black African) or else risk accounts circulating based on limited knowledge of the young individual with SCD, accounts not sanctioned by the young people themselves.

The wariness about the reaction of peers is not unfounded. B9 (Male, 18, Black Caribbean) and B11 (Male, 15, Black Caribbean) reported being physically punched or pushed respectively. B3 (Male, 25, Black Caribbean) was ‘bullied a few times because I was sick, yeah, that was really painful’. Another interviewee said:

B14: When you get sick in the classroom, and you get yourself worked up about it, they don’t understand what is happening to you. They think you are faking it (.) it is not good. They say things about you (.)[*Interviewer: What did they say?*]

B14: Things like, ‘You’re the one with yellow eyes, you have sickle, sickly … and hurtful stuff like that. (Male, 17, Black African)

B14 reports having to endure a combination of discriminatory attitudes: disbelief in SCD pain; derogatory comments about jaundice; and a linguistic play on the word sickle: a poignant piece of discrimination since emotional upset is itself a recognised trigger for provoking sickle cell illness.

As we have seen with respect to the school field, school absences and difficulties of school re-entry make a young person with SCD vulnerable to social isolation. Respondent B11 recounts how his mother has to stand up for him ‘once a week’, but that the teachers ‘don’t listen’ because ‘they don’t like certain pupils’. Asked why this was, he replied:

B11: Erm, erm, they erm, they say I was hanging around with one of my friends and my teacher didn’t like that one, he wouldn’t like me because I’d be hanging around with him, and I might be, like, the same as him so if he doesn’t like him, he won’t like me. (Male, 15, Black Caribbean)

It transpires that the reasons given for this reaction to pupils are that they ‘talk back’ and ‘don’t listen’, the teacher interpreting behaviour in the context of other discursive practices that permeate the world of the school. In this instance B11 has to navigate a tenuous position between being isolated through his school absences and being accepted by a peer group. Because his peer groups are regarded as a negative influence he is attributed the same negative qualities by association. He then thinks this is a reason why his mother’s constant advocacy regarding his SCD fails to change his school experience.

This is but one of several ways in which simply providing schools with information about sickle cell does not necessarily lead to improvements in the lived experiences of pupils with SCD. Overall, information to the effect that a child has SCD makes no appreciable difference to how well they feel they are treated by others at school (Dyson *et al.* 2010b). For example, one young woman states:

G2: Yeah, well I told [the teacher], but I don’t think she understood what that [sickle cell] was, so yeah, she just ignored it, she thought it was something minor. When I was coming back, it was an ambulance waiting for me at school. Then mum and everyone went to the hospital. And that was the first time I had blood transfusion, yeah, because I was really ill. And I think I was there for 4 weeks, and that was when they kind of realised that it was not something minor. (Female, 15, Black Caribbean)

Information about SCD is apparently disbelieved in the sense that the serious nature of the unseen condition is not appreciated. The variability of the condition, the numerous systems and the extreme pain experienced are also disbelieved. Moreover, information degrades: it may not be transferred at key points in the working life of the school.

Since medical strategies often do not work, the young person is faced with great challenges in constructing tactics for living with SCD at school. We have seen that the fields of the clinic and the school pull in opposite directions. The dispositions that the young person with SCD adopts are vulnerable to unwanted disclosure, to stigma, to exclusion, even to bullying. They appear to have no grand strategy that would enable them to satisfy the pressures of clinic and school, of teachers and peers, of health preservation and self preservation. Consequently, their dispositions are local, transient and vulnerable. The tactical emotional labour of mothers, in trading their own and their child’s current emotional capital for future educational capital (Reay 2004b) is less possible here, as draining emotional capital itself renders the young person with SCD corporeally vulnerable.

The fragility of their tactics derives also from the manner in which habitus is both structured and experienced as unique, with their dispositions highly sensitive to context. Reay (2004a) suggests the dispositions of habitus comprise several matrices: embodiment; agency; past/present and collective/individual. These matrices affect individual habitus in ways that create the sense of the uniqueness of individual experience. First, habitus is about how the social is in the body. B11 (Male, 15, Black Caribbean) contorted and cramped his long adolescent limbs to ride into the room on a child’s bike several sizes too small (possibly not updatable on the finances of his low-income single mother with 10 children), but sat for the interview legs akimbo, slouched back, arms draped wide: a posture likely to be read as retaliatory symbolic violence if displayed at school. Secondly, agency may stem from a dialogue of the ‘me’ (defined by others) and the self-determining ‘I’ (Shilling 2004). Thus the careers teacher earmarked bricklaying for B11, though he aspired to be an artist.

Thirdly, as with Kenway and McLeod’s (2004) low-income women, in contradiction of his past constraints of low teacher expectations, at present the young man says ‘sometimes I think of like staying on [at school, beyond minimum school leaving age]’. Finally, although B11 collectively shares poverty and a single mother upbringing with some other Black working-class students, he has an individual life history where ‘my oldest brother, he’s basically like a daddy to me … what a dad should be doing, he’s basically that’, so now his older brother is no longer at school to challenge teachers and advocate on behalf of B11, the absence of emotional labour by an older male on his behalf is keenly felt. Thus, as Reay (2004a) claims, his orientation is the product of a matrix drawing on both collective (class) experience and individual (life history) experience.

The fragility of these dispositions is further mirrored in the difficulties in transforming their fields of experiences. One way in which their experiences could be transformed is through the enactment of robust school policies. We encountered a number of innovations in the course of the overall fieldwork for the project, relatively rare occasions when a school policy supported the young person with SCD. One school devised a special card to permit toilet privileges; another operated a learning advice centre in twilight hours to enable all children who had missed lessons to catch up in the presence of a teacher; yet another changed school policy to allow all young people to wear long trousers rather than shorts and timetabled the class with a young person with SCD away from classrooms known to be cold. But invariably these were enacted in isolation and there was little sense that all such initiatives were required simultaneously.

Meanwhile, as reported elsewhere (Weiss *et al.* 2003), mothers here played a substantial role in informing, negotiating, mediating and advocating on behalf of their children. The information that their child had SCD had to be repeatedly given to schools, as it was not passed on when the young person changed classes and changed school, and was rarely passed on to or was rarely apprehended or acted upon by supply teachers. Mothers asked for work, collected work, helped with work and drove for hours at weekends so their children could attend fee-paying supplementary education. They sometimes had to co-opt the written evidence of a hospital consultant or the physical presence of a specialist sickle cell worker (where available) as an authoritative warrant of their child’s illness. In return they risked being labelled aggressive or telling teachers how to do their jobs, or their assertiveness was taken as evidence of family dysfunction.

Throughout, reactions to SCD remain as a potential negative resource for the young person. In Bourdieu’s terms, these reactions may be experienced as a form of negative capital: they drain somatic capital through painful episodes, lethargy, jaundice, and reduced body weight, they undermine cultural capital, whether it is institutionalised in educational qualifications or embodied through inclusion in the curriculum and broader school life, and they negate social capital by disrupting valued peer networks and valuable relationships of influence and support with teachers.

These three processes: isolated school policy innovations; the mother as mediator and advocate; and the drain on various forms of capital represented by the orientation of others to SCD frame the school experiences of young people with SCD: habitually positioned between contradictory fields of clinic and school and left with only fragile tactics to employ in negotiating school lives.

## Capital gained, capital lost

The fact that some young people with SCD succeed in education is viewed by them as luck in managing their condition (B5, Male, 25, Black African) and in this sense SCD arguably masks structured experiences. For Bourdieu, class is the meta-field. Those who fared better were middle rather than working class. B18 (Male, 17, Black Caribbean) had attended a series of international schools. His father had a professional job with an international agency and ‘I was out of school for a while because dad had to stop work and stay in the hospital with me’, a job flexibility not usually extended to those in manual occupations. He had obtained ten General School Certificates of Education, having entered the UK education system only at the age of 15 (see Gillborn [2008] on the inverse relationship between time spent in the British education system and the achievement of Black pupils), and wanted to try acting but ‘if not I want to fall back on law and do, like, a [Common Professional Examination] conversion test and then go into that’. By contrast to the law as a second career choice, the aspirations of B3 (Male, 25 Black Caribbean) were more modest. Having alleged a lack of support at four different further education colleges, he stated simply: ‘hopefully, I want to work’.

B10 (Male, 11, Black African, accompanied at the interview by his father) already had aspirations to attend university. He continually mentioned ‘Mum or Dad’ being contacted by the school, parents (plural) bringing in medication to school, and ‘if I don’t feel well my *Mum or Dad* [our emphasis] will write a note for me to take in’ to school. Although he has a form of SCD said to be clinically severe (haemoglobin SS), his nurse counsellor attributed his good health to having two wage-earning professionals as parents. B11 (Male, 15, Black Caribbean) had the ostensibly less severe haemoglobin SC, but his nurse counsellor attributed his much higher levels of illness to the low income of a family headed by a single mother of 10 children. In Bourdieusian terms, his class position undermined his health capital, leading to extensive school absences, further undermining his already weak accumulation of educational capital.

Those who progressed to university enjoyed a positive school ethos, in which, for example, bullying was challenged not ignored. B13, who was currently at university, recounted:

B13: Someone pushed me at school once and I fell and hurt my back and started getting pains and my parents were obviously furious and they contacted the school and the head of year called in at the student’s parents and they had to get told what he had done, and then that guy was OK with me. (Male, 21, British Asian)

By contrast G7, who was not expecting to attend university said:

G7: They [the teachers] were like, ‘Oh wait till, wait to do whatever you need to do till the end of the lesson’ or ‘[Name], be quiet’. All I am saying is ‘I feel sick and if I walk out now you’ll be the one to complain so I am telling you I feel sick so you can help me do something’, and then it was mostly, it was mostly, ‘Oh not again’ sort of thing, you know, that sort of sigh. Sighs, ‘Here we go again’ sort of thing. And it was frustrating for them, I guess, but more so for me and I was the one that mostly depressed. It was worse for me because I was going through it. Every time you battle with the teachers you went straight to battling with the students. I never got any peace from anywhere. It was all right Year 7 to Year 8 and then Year 9 came and Year 10 came and Year 11 came and I just wanted to hurt everyone, especially someone who had something negative to say and that was the first thing they would bring up. The minute you had a disagreement with someone, the minute you argued with someone, the first thing they would say to piss me off would be, ‘At least I don’t go hospital all the time. At least my second home ain’t a hospital’ or ‘At least I don’t get sick like a freak’, at least this or at least that. ‘At least I don’t have rainbow coloured eyes’, ‘At least I don’t glow in the dark’, basically my eyes; so on and so forth. The minute they mention it was the minute I got to the very, very end of my peak. Then we would have a fight ’cos I disliked that comment, I disliked all of those comments so much it was just, like, it was a way to set me off. It was a sure way to get me to say something to them and they would come for me and then we would get into a fight. I never actually hit first. I wouldn’t because then it would be all my fault but if they hit first but then I said something it would be both our faults. … I think that is the main reason why secondary I hated so much. (Female, 18, Black Caribbean)

She suggests that teachers regarded her recurrent bouts of extreme pain as troublesome to them and that peers used her illness as the site around which to taunt and bully her. She maintains she did not hit first and that even retorting with words was sufficient for the teachers to construe the event as one in which the fault was on both sides. The fact that G7 suggests the bullying ranged across several years of school and that her sickle cell happened to be the particular weakness picked on in her case, suggests that bullying was both endemic and ineffectively challenged through much of her secondary education.

G9, currently at university, recalled being included on a school trip abroad, where others managed to support her successfully through her painful sickle cell episode:

G9: In my last year, in my A-level year we had a trip to Spain, which was also for ten days. And I had a crisis there. That was the only time I had a crisis with flat mates with me. Apart from that I always had it at home or on holidays. But at the last day, unfortunately, I had this crisis and we had to drive back 17 hours in the bus. So that was very uncomfortable. The teacher saw sure that I had as much space as possible in a crowded bus. I had two chairs in the bus so I could lay down and relax a little bit to be comfortable. Everyone was very considerate. The whole grade was very considerate and they took great care of me. (Female, 20, Black African)

G1 was a wheelchair user and was excluded from schools trips, though the discourse of the head teacher constructs her exclusion as acts of kindness, anticipating problems and thoughtfully contacting the mother well in advance for her to make other arrangements:

G1: [Head teacher] he’d say, ‘Hi’ and I’d say, ‘Hi’ and he’d say, ‘[Name], we’re going on a trip next week: we’re having to tell your Mum duh duh’. And he would tell me in advance that I had to tell my mum so they could sort out what would happen and stuff. (Female, 16, Black African)

In summary, those who were successful in gaining educational capital, as evidenced by their actual or likely attendance at university, were more likely to be middle class, enjoy good levels of parental involvement in school liaison (and whose involvement was accepted by the school), enjoy effective intervention by the school in relation to bullying and benefit from an inclusive school ethos, as evidenced by participation in school trips.

## Conclusion

In this article we have considered the manner in which young people with SCD negotiate their lives at school, drawing upon the work of Bourdieu and his concepts of field, habitus and capital. Bourdieu was concerned with how an individual is simultaneously defined and realised through the processes of social negotiation and the specific context of power relationships. One principal field in which young people with SCD assume different positions is that of the clinic. They have to engage in a medical strategy using various forms of health capital derived from their physical attendance at medical sites for treatment and monitoring and the inculcation of care of the self in maintaining good hydration, moderating exercise, resting appropriately and regulating their temperature.

However, when struggling to have their view of the world regarded as legitimate within the field of school relations, young people with SCD are faced with considerable challenges. The school field requires its subjects to adhere to routines, not to disrupt classes with requests for water or toilet breaks, to follow rules in school attire and in being outside during break-times, to participate to one’s physical limit, to attend regularly, to concentrate and to achieve consistently. Thus medical strategies, which might give some legitimacy to these children’s behaviour, find little if any expression in the classroom. Teachers do not recognise the legitimacy of these children’s accounts and use their own power of definition to impose other interpretations on the child’s behaviour, such as laziness, and occasionally these dovetail with wider gendered and racist assumptions. As a result, young people with SCD are dys-positioned by two fields of relations habitually at odds with one another. However, the outcomes are not guaranteed but subject to ongoing negotiation. The struggle through dispositions creates the potential that relationships can be challenged and altered rather than ‘imprisoning’ agency ‘in a negative essence’ (Bourdieu 1990: 28). Individuals, as part of active agency, are able to use norms, attitudes and beliefs to define who they are, as part of a negotiated relationship.

But a key strategy endorsed by health professionals, namely that of providing information that they have SCD to teachers and fellow pupils, plays out poorly as a tactic: it works partially, inconsistently or even worsens the school experience. Faced with such complexity, the tactics for living adopted by young people with SCD are contingent and tentative. The habitus they create is reduced to a series of tentative dispositions. Sometimes trusted dispositions prove perverse and entirely undermine the place of the young person with SCD in the world.

More structural aspects associated with these norms and values can impose themselves too. Their fragile dispositions are bounded by three external series of actions. First, mothers mediate the experience of their children, struggling to find the right balance of advocacy and supplication that will ameliorate the lives of their loved ones. Secondly, policy innovations to support the young person are in evidence, but while a school may have developed a policy on one dimension of relevance, in no instance was this reported to be linked to policies addressing other dimensions of need. Finally, young people were aware that reactions to their SCD continually constituted a potential net drain on their cultural, somatic and social capital. Self-actualisation for young people with sickle cell disorder, therefore, involves both collectivity and individuality (Bourdieu and Wacquant 1992) in which their lives are structured. They enact their lives in nuanced ways but not everything is possible. Such tensions remain at the heart of the narratives of young people with sickle cell.
